# Factors Influencing Asthma in Children at Early Childhood Development Centres in a Densely Populated Urban Informal Township in Gauteng Province, South Africa

**DOI:** 10.3390/children13050627

**Published:** 2026-04-30

**Authors:** Velisha Thompson, Joyce Shirinde, Masilu D. Masekameni, Thokozani P. Mbonane

**Affiliations:** 1 Department of Environmental Health, Faculty of Health Sciences, University of Johannesburg, Johannesburg 2001, South Africa; maseksd@unisa.ac.za; 2 School of Health Systems and Public Health, Faculty of Health Sciences, University of Pretoria, P.O. Box 667, Pretoria 0001, South Africa; joyce.shirinde@up.ac.za; 3 Department of Development Studies, School of Social Sciences, College of Human Sciences, University of South Africa, Pretoria 0003, South Africa

**Keywords:** chronic inflammatory respiratory conditions, environmental factors, asthma, wheeze, low- and middle-income countries, vulnerable communities, household factors, environmental health

## Abstract

**Highlights:**

**What are the main findings?**

- The prevalence of asthma is high among children 5 years or younger in urban informal settlements.
- The occurrence of asthma and wheeze is influenced by individual and environmental risk factors among children 5 years or younger in urban informal settlements.

**What are the implications of the main findings?**

- There is a need for a holistic approach to addressing preventable factors influencing the prevalence of asthma.

**Abstract:**

Background: Asthma is one of the leading chronic inflammatory respiratory conditions affecting children under 5 years of age, especially those who reside in socio-economically disadvantaged and densely populated low- and middle-income communities. Methods: A cross-sectional analytical study was conducted to ascertain the prevalence of factors influencing asthma and wheeze among young children attending early childhood development centres in Alexandra Township. Data were collected using a self-administered modified International Study of Asthma and Allergies in Childhood questionnaire. The analysis was performed utilising STATA version 19. The study sample comprised 3265 young children and their parents or guardians. Results: The findings reveal that the prevalence of asthma and current wheeze was 17.52% and 35.56%, respectively, while the prevalence of a history of wheeze was 64.36%. In the multivariate analysis, a family history of asthma was identified as a risk factor for asthma (*p* < 0.001) and for current wheeze (*p* < 0.001) and historical wheeze (*p* < 0.001). Additionally, the use of pain medication and passing of public transport were seldom identified as risk factors for both asthma and wheeze. Furthermore, exposure to second-hand tobacco smoke (*p* = 0.025) was found to influence the occurrence of asthma. Conclusions: This study highlights the impact of individual, household, and environmental factors on asthma. The findings are critical for the implementation of preventive environmental health measures to address this issue, particularly in low- and middle-income countries with limited curative resources.

## 1. Introduction

Asthma, a chronic inflammatory respiratory condition, is a common condition among young children, especially asthma characterised by wheeze, difficulty breathing, and persistent coughing [1,2,3,4]. The impact of asthma is well researched globally, with an estimated prevalence of over 260 million individuals, and a higher prevalence in high-income countries. However, there is a paucity of information on the mortality, morbidity, and determinants of this condition in the sub-Saharan African region [5,6]. Yet, asthma is the most reported cause of chronic respiratory deaths [7]. This condition is triggered by numerous preventable individual, environmental, and household risk factors [5,8,9].

The prevalence rate of asthma among children in Africa is estimated to be around 11% to 21.90%, and it has been increasing in the last four decades [10,11]. This is a much higher rate than that of Europe (9–11%), Asia (10%), and the United States (8.3%), but it is similar to that of South American countries (20–30%) [11,12,13]. However, the reported prevalence rates of asthma in the sub-Saharan African region may not accurately reflect the true extent of the condition, as there is a paucity of scientific evidence regarding this phenomenon [14]. Furthermore, many of the environmental and household triggers of asthma that remain persistent issues in the sub-Saharan African region have largely been overlooked.

Asthma is triggered by a complex interplay between genetic and environmental risk factors. Genetic predisposition may encompass a family history of asthma and related allergic conditions, as well as an impaired immune response. By contrast, environmental risk factors are diverse and include proximity to high-traffic areas, utilisation of polluting fuels, ownership of a domestic animal (particularly a dog or a cat), and influences from climate change. Additional environmental contributors include seasonal weather fluctuations, exposure to chemical pollutants, and second-hand smoke [15]. Studies conducted among children in low- to middle-income countries have highlighted the significant association between asthma and wheeze and exposure to second-hand smoke [15,16,17].

This study was conducted in Alexandra Township, an impoverished informal settlement with a myriad of health challenges, situated in the north of the City of Johannesburg, Gauteng Province, South Africa. Alexandra, a densely populated urban informal township with narrow roads, serious housing shortages, and a high crime rate, is separated by a highway from the affluent suburb of Sandton, a financial hub of Africa [18]. Continuous environmental and domestic pollution are significant contributors to asthma and respiratory conditions in urban settlements [12,19,20]. To our knowledge, this is the first cross-sectional analytical study investigating the prevalence of asthma and its associated risk factors in Alexandra, focusing on children aged 5 years and younger at early childhood development (ECD) centres.

## 2. Materials and Methods

### 2.1. Study Design and Setting

A cross-sectional analytical study was conducted from April 2024 to June 2025 at ECD centres in Alexandra, a densely populated urban informal township (bordering Jukskei River and the adjacent industrial area) located in the City of Johannesburg, Gauteng Province, South Africa. The township has an area of approximately 8 km^2^ and was originally designed to accommodate a maximum of 30,000 residents. However, the current population of Alexandra is estimated to range between 750,000 and 1,000,000. The majority of residents in this densely populated township reside in slums and informal backyard dwellings, with some lacking access to formal municipal services, such as electricity, water, and sanitation. This situation has a significant negative impact on the environment [21,22,23].

### 2.2. Study Population, Selection Criteria, and Sampling

The targeted study population comprised children aged 5 years or younger and their parents or guardians. The study included children who had resided in the area for over 12 months and who were enrolled in early childhood education programmes in Alexandra. Children living with a parent or guardian who was mentally ill, seriously ill, or under the age of 18 were excluded from participation in the study. We estimated the study population based on the 162 identified ECD centres. The sample size was calculated using Epi Info 7.2; however, we aimed to target a population of 3000 or more to adhere to the recommendations given in the International Study of Asthma and Allergies in Childhood (ISAAC) manual, to mitigate the risk of selection bias [24]. We randomly selected ECD centres, and those that granted gatekeepers permission were included in the study. Thus, every second child was randomly selected and approached for participation, contingent upon obtaining parental consent [25]. This process was repeated until a sample size of 3265 was reached.

### 2.3. Prevalence of Asthma and Wheeze (Current and Historical)

Asthma was defined based on affirmative responses to items in the written ISAAC questionnaire [24,26,27,28]. The ISAAC asthma definition was adapted from the European Respiratory Society (ERS) definition, as it serves primarily as a standardized epidemiological tool, whereas the ERS definition emphasizes clinical classification and management [29,30].

The responses were self-reported by parents or guardians of participating children. Core items addressing asthma-related symptoms were used to define specific outcomes, as follows:

- The outcome “asthma” was defined as a positive response to the question “Has your child ever had asthma?”
- The outcome “current wheeze” was defined as a positive response to the question “Has your child experienced wheezing, or whistling in the chest, in the past 12 months?”
- The outcome “history of wheeze” was defined as a positive response to the question “Has your child ever experienced wheezing, or whistling in the chest, any time in the past?” (The current wheeze and history of wheeze are the same for children aged 0 to 12 months).

### 2.4. Study Risk (Household and Environmental) Factors

We first collected data on socio-demographic variables, including the child’s gender (male/female), the child’s age (12 months or younger/13–36 months/37 months or older), duration of residence in the township (less than 1 year/1 to 3 years/3 years or longer), family history of asthma (no/yes), mother or female guardian’s smoking status (no/yes), father or male guardian’s smoking status (no/yes), and use of pain medication, such as aspirin or paracetamol (never/occasionally/frequently). Then, we collected data on household and environmental risk factors, such as access to running water (no/yes), ownership of an indoor domestic cat (no/yes), ownership of a domestic dog (no/yes), and frequency of public transport passing near the residence (never/seldom/frequently throughout the day/almost all day). We created the following variables using the data collected: dirty (wood, coal, or paraffin) or clean (electricity or gas) fuels for cooking and heating, and exposure to second-hand tobacco smoke (ETS) within the household was assessed based on the smoking status of parents or guardians. The presence of either a maternal or paternal guardian who smokes was classified as an indicator of ETS exposure (no/yes).

### 2.5. Data Management and Analysis

The primary data were recorded, cleaned, coded, and captured in a Microsoft Excel spreadsheet, which was then exported to the latest version of the STATA 18 software for analysis. To determine the risk factors for asthma in the study, a multivariate logistic regression model was used. First, a bivariate binary logistic regression analysis was conducted between asthma (the dependent variable) and all the socio-demographic characteristics, the environmental risk measurements, and the household and ECD centre risk factors (the independent variables). Household conditions that were deemed to contribute to exposure included the type of house (formal/informal), owning pets/animals, environmental tobacco smoke exposure at home, and the type of fuel used for cooking and heating. Other potential exposure or confounding factors were time lived in the area (<3 years/≥3 years), the location of the home and the ECD centre, proximity to major/busy roads, and the health status of the child. All the bivariate analyses that showed a significant association were included in the report. To evaluate the likelihood of health consequences given the presence of confounding variables and the source of air pollution, crude odds ratios and 95% confidence intervals were calculated. In the multivariate logistical regression, only *p*-values below 0.05 were deemed statistically significant.

## 3. Results

### 3.1. Participants’ Socio-Demographic Characteristics

The demographic characteristics of the study population (n = 3265) are depicted in Table 1. Females constituted the majority of the participants, representing 53% of the total, while males comprised 47%. The majority of the participants were aged 37 months or older. A significant proportion (38%) reported having resided in the township for over three years. A family history of asthma was indicated by 41.6% of the participants. Four-hundred-and-ten children (12.6%) stayed with a mother or female guardian who smoked, while 760 children (23.3%) stayed with a father or male guardian who smoked. The majority of the participants never used pain medication (80.5%), while 19.6% used it occasionally or frequently.

### 3.2. Prevalence Rates for Asthma

**Figure 1:**
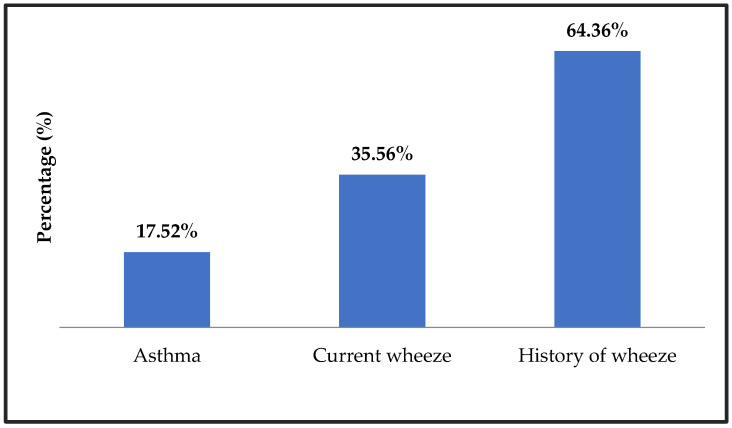
The prevalence of asthma, current wheeze, and history of wheeze.

The prevalence rates for the three health outcome variables were as follows: 17.52% for asthma, 35.56% for current wheeze, and 64.36% for history of wheeze, as shown in Figure 1. The prevalence was found to be higher for male children (16.78%) than for female children (15.23%). When stratified by age group, the prevalence rates were as follows: less than 1 year (12.54%), 1 to 3 years (15.09%), and 3 years or older (17.52%).

### 3.3. Household and Environmental Risk Factors

Table 2 shows that 57.5% of the study participants had access to running water, while 42.5% did not have access. Although lack of running water is not a direct source of environmental air pollution, it may indirectly increase susceptibility to respiratory illness due to poor hygiene and a higher risk of infections. The results also show that the majority of the study participants used electricity for cooking and heating purposes. Small proportions of the participants reported using gas for cooking purposes (11.5%) and for heating purposes (3.4%). Dirty fuels (wood, charcoal, kerosene, etc.) were used by 13.4% of the participants, while 86.6% used clean fuels. Paraffin (4.5% for heating, 2% for cooking) and open fires (0.35%) for heating were used by small proportions. While 45.3% of the participants were not exposed to motor vehicle pollution, almost 30% were exposed frequently or continuously.

### 3.4. Association Between Asthma Prevalence and Socio-Demographics and Risk Factors

#### 3.4.1. Bivariate Relationship Between Asthma, Current Wheeze, and History of Wheeze and Socio-Demographics and Risk Factors

Table 3 summarises the bivariate relationship between asthma, current wheeze, and history of wheeze and individual socio-demographics and environmental and household factors for the study population. Asthma showed a higher prevalence in the age categories of 13–36 months and 37 months or older (COR = 1.394 and COR = 1.436, respectively). For the age category of 13–36 months, current wheeze had a crude odds ratio of 1.486 (*p* = 0.003), which was statistically significant. The age category of 12 months or younger showed no significant association with wheeze and asthma. Having a family history of asthma increased the odds of asthma (COR = 2.748), current wheeze (COR = 2.400), and history of wheeze (COR = 2.606). A significant association was observed between parental (mother and father) tobacco smoke and asthma (COR = 2.315 and COR = 2.346, respectively). Similarly, a significant association was observed between the use of pain medication occasionally and frequently and asthma (COR = 2.055 and 2.883, respectively). Dirty fuels used for cooking and heating purposes increased the likelihood of wheeze (COR = 3.939 and COR = 2.243, respectively). Having a cat at home increased the likelihood of asthma (COR = 1.728) and history of wheeze (COR = 1.512).

#### 3.4.2. Factors Associated with Asthma, Current Wheeze, and History of Wheeze

In the multivariate analysis, several variables were associated with wheeze (see Table 4). The age category of 13–36 months (AOR = 0.485, *p* < 0.001), a family history of asthma (AOR = 2.450, *p* < 0.001), and lack of running water (AOR = 1.252, *p* < 0.001) were associated with a history of wheeze. Use of pain medication occasionally (AOR = 3.135, *p* < 0.001) and frequently (AOR = 2.974, *p* < 0.001) and residing on a street with “seldom” public transport passing (AOR = 1.672, *p* = 0.004) were associated with asthma.

## 4. Discussion

The study found a disturbingly high burden of asthma and related respiratory symptoms among children attending ECD centres in Alexandra, with prevalence rates exceeding global averages and reflecting trends reported in other African urban settlements.

### 4.1. Prevalence of Asthma and Associated Risk Factors in the Study

The asthma prevalence of 17.52% observed in this study is higher than that for European and Asian children [31,32]. In a longitudinal study conducted in Spain, Alfonso and colleagues reported a 12.8% prevalence of asthma [32]. A cross-sectional study conducted in China among children aged 3 to 6 years reported an asthma prevalence of 11.2% [31]. However, the asthma prevalence in the current study falls within the reported range (1.70–20.85%) of asthma prevalence in sub-Saharan Africa [30]. The observed difference in prevalence may be attributable to a well-developed infrastructure, which mitigates environmental risk factors as well as improves access to primary healthcare services, in high-income countries that are better resourced [33,34]. These environmental risk factors can be managed or controlled to address the triggers that contribute to the increase in asthma cases. Lastly, male children and older children exhibited higher prevalence rates in this study, a finding consistent with research conducted in other contexts [35,36,37].

Dominant risk factors associated with childhood asthma include a family history of the condition, exposure to second-hand smoke, maternal educational attainment, male gender, delivery by caesarean section, the presence of rhinitis and eczema, ownership of a domestic cat or dog, residence in proximity to roads with a high density of traffic, a lack of breastfeeding, consumption of fast food, use of pain medication, and exposure to environmental pollution, particularly air quality issues [11,29]. In the current study, a family history of asthma, use of pain medication, residing on a street with “seldom” public transport passing, and exposure to second-hand smoke were identified as significant risk factors for asthma. These findings are similar to those of studies conducted elsewhere, as reported in the systematic review and meta-analysis [11]. Indoor factors, including solid fuel use, second-hand smoke, and allergens, alongside outdoor factors such as traffic volume, emissions, particulate matter, and ground-level ozone, constitute significant environmental risk factors for pediatric asthma [38,39,40]. These risk factors are common triggers of new asthma cases and exacerbations, particularly in low- and middle-income countries [38,41,42]. In this study, exposure to second-hand smoking and residing on a street with “seldom” public transport passing were identified as air pollution risk factors. A recent study in South Africa, Mpumalanga, found that having someone as a smoker at home was a trigger for asthma among preschool children [17]. Furthermore, a study conducted by Gasana et al. found that children residing in proximity to roads with a high density of traffic, or attending schools in such areas, are exposed to elevated levels of motor vehicle air pollutants, which contribute to an increased incidence and prevalence of childhood asthma [43]. These risk factors are manageable and preventable through proactive environmental health programmes.

### 4.2. Prevalence of Current Wheeze and History of Wheeze and Associated Risk Factors in the Study

Approximately 50% of children under the age of 6 experience wheezing at some point in their development [44,45]. This includes transient viral wheeze, normally identified as a “whistling noise” while a child is experiencing an episode of a cold or similar virus. The prevalence of a history of wheeze (64.36%) was significantly higher than that found in a recent study (15.14%) conducted among preschool children residing in both rural and urban areas of a low- and middle-income country [17]. This could be attributable to over-reporting or misinterpretation by parents during data collection, as they might have reported on a transient viral wheeze episode that occurred in the past [46,47,48]. However, a high prevalence of history of wheeze (53%) has been reported in Turkey in a case–control study involving preschool children [49]. The prevalence of current wheeze in this study was in the same range as that found in studies conducted in an urban area [49]. The elevated prevalence of historical wheeze and current wheeze may be associated with various environmental triggers. Current wheeze was found to be significantly related to several factors, including age (specifically older age), duration of residence in the township (exceeding three years), maternal and paternal smoking, a family history of asthma, ownership of a domestic cat, and proximity to areas with a high density of traffic, particularly those influenced by public transport. Similarly, historical wheeze was associated with age (again, older age), the number of years having resided in the township, a family history of asthma, and cat ownership. These factors have been documented in previous studies as contributors to asthma symptoms [11,29].

The findings of this study emphasise the need for urgent public health interventions focused on both mitigating environmental exposure and strengthening healthcare infrastructure within informal settlements. Recommended strategies include promotion of cleaner fuels, reduction of ambient and indoor air pollution, improved water and home infrastructure, and better access to preventive healthcare for young children. The findings also highlight persistent environmental inequity and the need for policymakers to prioritise at-risk communities facing disproportionate asthma burdens. Future research should use longitudinal designs to explain causal pathways, explore gene–environment interactions, and test targeted interventions aimed at reducing child respiratory morbidity within similar low-resource urban contexts. Coordinated approaches integrating health, housing, and environmental policy will be essential for sustainable change.

### 4.3. Strengths and Limitations

The main strength of this study is its utilisation of a validated data collection tool, namely the ISAAC questionnaire, which has been employed across diverse contexts and age groups of children. The questionnaire is ideal for asthma screening, as clinical diagnosis is not reliable for the age group of 5 years or younger. Additionally, adherence to the recommended sample size exceeding 3000 participants mitigates the risk of selection bias. However, one notable limitation is the cross-sectional design of the study, which precludes the ability to investigate causal relationships related to the disease. In addition, the absence of clinical asthma diagnoses and the reliance on self-reported data from the questionnaire may introduce recall bias or misrepresentation of health outcomes among participants, potentially leading to misclassification of asthma and wheeze (current and historical) prevalence.

## 5. Conclusions

This study highlights that the prevalence of asthma and wheeze is still higher in children residing in densely populated urban informal townships. Furthermore, household and environmental risk factors are triggers for asthma and wheeze in children at ECD centres. The study findings emphasise the urgent need for targeted interventions to mitigate exposure, improve urban infrastructure, and enhance access to preventive healthcare in high-risk communities. Greater prioritisation by policymakers and health professionals is essential to address these inequities and protect vulnerable populations. Longitudinal studies and intervention trials are necessary to explain causal pathways, refine prevention strategies, and monitor progress in curbing the childhood asthma epidemic in low-resource urban areas. Overall, multi-sectoral collaboration offers the best prospect for reducing environmental risks, improving childhood respiratory health, and achieving lasting improvements in urban well-being.

## Figures and Tables

**Table 1:** The study participants’ socio-demographic characteristics (n = 3265).

Socio-Demographics	Frequency (n)	Percent (%)
Gender		
Males	1538	47%
Females	1727	53%
Age		
12 months or younger	303	9.3%
13–36 months	1478	45.3%
37 months or older	1484	45.5%
Duration of residence in township		
Less than 1 year	1394	42.7%
1 to 3 years	630	19.3%
3 years or longer	1240	38%
Family history of asthma		
No	1907	58.4%
Yes	1358	41.6%
Mother/female guardian smoking		
Yes	410	12.6%
No	2855	87.4%
Father/male guardian smoking		
Yes	760	23.3%
No	2505	76.7%
Use of pain medication		
Never	2628	80.5%
Occasionally	263	8.1%
Frequently	374	11.5%

**Table 2:** Description of the study participants’ household risk factors.

Risk Factor	Frequency (n)	Percentage (%)
Running water		
Yes	1878	57.5%
No	1387	42.5%
Fuels used for cooking		
Dirty fuels	438	13.4%
Clean fuels	2827	86.6%
Fuels used for heating		
Dirty fuels	271	8.3%
Clean fuels	2994	91.7%
Cat		
Yes	444	13.6%
No	2821	86.4%
Dog		
Yes	452	13.8%
No	2813	86.2%
Public transport		
Never	1478	45.3%
Seldom	830	25.4%
Frequently throughout the day	624	19.1%
Almost all day	333	10.2%
Exposure to second-hand tobacco smoke		
Yes	870	26.6%
No	2395	73.4%

**Table 3:** Bivariate analysis of asthma, current wheeze, and history of wheeze with socio-demographics and risk factors.

Socio-Demographics		Asthma		Current Wheeze		History of Wheeze	
		COR	*p*-Value **	COR	*p*-Value	COR	*p*-Value
Gender	Male	Ref					
	Female	0.931	0.426	1.105	0.169	0.837	0.018 *
Age	12 months or younger	Ref					
	13–36 months	1.394	0.059	1.486	0.003 *	0.460	<0.001 *
	37 months or older	1.436	0.039 *	0.949	0.694	0.419	<0.001 *
Duration of residence in township	Less than 1 year	Ref					
	1 to 3 years	0.930	0.554	0.586	<0.001 *	1.080	0.467
	3 years or longer	0.962	0.699	0.447	<0.001 *	0.750	0.001 *
Family history of asthma	No	Ref					
	Yes	2.748	<0.001 *	2.400	<0.001 *	2.606	<0.001 *
Mother/female guardian smoking	No	Ref					
	Yes	2.315	<0.001 *	1.753	<0.001 *	1.090	0.448
Father/male guardian smoking	No	Ref					
	Yes	2.346	<0.000 *	1.429	0.000 *	1.047	0.602
Use of pain medication	Never	Ref					
	Occasionally	2.055	<0.001 *	0.467	<0.001 *	0.388	<0.001 *
	Frequently	2.883	<0.001 *	0.144	<0.001 *	1.438	0.004 *
Running water	No	Ref					
	Yes	1.136	0.161	0.597	<0.001 *	0.332	<0.001 *
Cooking fuels	Clean fuels	Ref					
	Dirty fuels	1.016	0.906	3.939	<0.001 *	0.771	0.023 *
Heating fuels	Clean fuels	Ref					
	Dirty fuels	0.718	0.027 *	2.243	<0.001 *	1.087	0.533
Cat	No	Ref					
	Yes	1.728	<0.001 *	0.916	0.406	1.512	<0.001 *
Dog	No	Ref					
	Yes	1.502	0.001 *	1.118	0.308	1.451	<0.001 *
Public transport	Never	Ref					
	Seldom	0.778	0.025 *	0.523	<0.001 *	1.098	0.317
	Frequently throughout the day	0.679	0.003 *	0.520	<0.001 *	1.055	0.599
	Almost all day	0.941	0.6688	0.304	<0.001 *	0.770	0.307
Exposure to second-hand tobacco smoke	No	Ref					
	Yes	2.468	<0.001 *	1.397	<0.001 *	1.057	0.511

** *p*-value was set at 0.05 for statistical significance; * Statistically significant.

**Table 4:** Multivariate analysis of asthma, current wheeze, and history of wheeze with socio-demographics and risk factors.

Socio-Demographics		Asthma		Current Wheeze		History of Wheeze	
		AOR	*p*-Value **	AOR	*p*-Value	AOR	*p*-Value
Gender	Male	Ref					
	Female	1.008	0.931	1.133	0.114	0.866	0.066
Age	12 months or younger	Ref					
	13–36 months	1.269	0.217	1.361	0.034 *	0.485	<0.001 *
	37 months or older	1.295	0.200	1.218	0.199	0.456	<0.001 *
Duration of residence in township	Less than 1 year	Ref					
	1 to 3 years	0.989	0.941	0.863	0.199	0.940	0.601
	3 years or longer	1.027	0.844	1.707	0.002 *	1.757	0.011 *
Family history of asthma	No	Ref					
	Yes	2.745	<0.001 *	1.437	<0.001 *	2.450	<0.001 *
Mother/female guardian smoking	No	Ref					
	Yes	1.186	0.329	1.849	<0.001 *	0.871	0.418
Father/male guardian smoking	No	Ref					
	Yes	1.200	0.469	1.825	0.014 *	1.084	0.749
Use of pain medication	Never	Ref					
	Occasionally	3.135	<0.001 *	0.561	<0.001 *	1.467	0.067
	Frequently	2.974	<0.001 *	0.243	<0.001 *	1.102	0.486
Running water	No	Ref					
	Yes	1.118	0.259	0.678	<0.001 *	1.252	<0.001 *
Cooking fuels	Dirty fuels	Ref					
	Clean fuels	1.563	0.500	2.126	0.074	0.900	0.420
Heating fuels	Dirty fuels	Ref					
	Clean fuels	0.962	0.168	1.370	0.065	1.064	0.683
Cat	No	Ref					
	Yes	1.150	0.297	1.689	0.004 *	1.389	0.011 *
Dog	No	Ref					
	Yes	0.962	0.787	1.045	0.722	1.030	0.814
Public transport	Never	Ref					
	Seldom	1.672	0.004 *	1.704	0.001 *	1.96	0.964
	Frequently throughout the day	0.467	0.278	0.976	0.843	1.032	0.811
	Almost all day	0.143	0.251	0.457	0.211	1.060	0.799
Exposure to second-hand tobacco smoke	No	Ref					
	Yes	1.880	0.025 *	1.672	0.142	1.873	0.509

** *p*-value was set at 0.05 for statistical significance; * Statistically significant.

## Data Availability

The original contributions presented in this study are included in the article. Further inquiries can be directed to the corresponding authors, and the request must be made in accordance with the PoPI Act.
